# Roles of exosomes as drug delivery systems in cancer immunotherapy: a mini-review

**DOI:** 10.1007/s12672-022-00539-5

**Published:** 2022-08-13

**Authors:** Zhen Fang, Yixuan Ding, Zhigang Xue, Peijuan Li, Jia Li, Fei Li

**Affiliations:** 1 Department of General Surgery, Xuanwu Hospital of Capital Medical University, Beijing, 100053 China; 2 Dalian Medical University, Dalian, Liaoning China

**Keywords:** Exosomes, Drug delivery systems, Cancer, Immunotherapy

## Abstract

Exosomes can be released by a variety of cells and participate in intercellular communication in many physiological processes in the body. They can be used as carriers of cancer therapeutic drugs and have natural delivery capabilities. Some biologically active substances on exosomes, such as major histocompatibility complex (MHC), have been shown to be involved in exosome-mediated anticancer immune responses and have important regulatory effects on the immune system. Exosome-based drug delivery systems hold great promise in future cancer immunotherapy. However, there are still substantial challenges to be overcome in the clinical application of exosomes as drug carriers. This article reviews the biological characteristics of exosome drug delivery systems and their potential applications and challenges in cancer immunotherapy.

## Introduction

The term “exosomes” first appeared in the 1980s. Trams et al. discovered a set of vesicle-like structures with diameters ranging from 40 to 1000 nm using transmission electron microscopy [1]. Later, Johnstone isolated these vesicles from sheep reticulocytes by ultracentrifugation at 100,000×*g* for 90 min, and these vesicle-like structures were called exosomes for the first time [2].

The extracellular vesicles (EVs) collectively refers to various vesicles with membrane structures released by cells. Due to their different sizes and how they are formed, they are divided into three subgroups: exosomes, microvesicles, and apoptotic bodies (Table 1). Exosomes, also known as intraluminal vesicles (ILVs), are approximately 40–160 nm in size and are produced by inward budding of multivesicular endosomes (MVEs) during maturation [3, 4]. Cells release exosomes after MVEs fuse with the cell membrane. The endosomal sorting complex (ESCRT), Rab protein, CD36 and sphingolipid ceramide required for the transport mechanism have been shown to play important roles in biological processes [5–8]. Exosomes can be secreted under physiological and pathological conditions by almost all types of cells, including prokaryotic cells and eukaryotic cells [9, 10]. They are widely present in culture supernatants and biological fluids such as blood, urine, breast milk, pleural fluid and cerebrospinal fluid. The exosomes released from one cell type (donor cells) can be taken up by another (recipient cells). If release and uptake of exosomes are arisen by same cells, it is called to be autologous (or autocrine). If these actions are achieved between different or remote cell types, it is called to be heterologous (or paracrine) [8, 11].

**Table 1:** Classification of extracellular vesicles

Characters	Exosomes	Microvesicles	Apoptotic body
Size	40–160 nm	100–1000 nm	100–5000 nm
Markers	LAMP1, Tetraspanins, Alix, MHC I/II, HSP70, TSG100	Selectins, integrins, tissues factors and cell-specific markers	Histones, organelles
Origin	Endosomal compartments of cells	Cell surface plasma membrane	When cells undergo apoptosis

In recent years, the concept of precision medicine has been widely accepted. To improve the therapeutic effect of drugs and reduce toxicity and side effects, researchers have gradually begun to pay attention to precise and efficient drug delivery systems. Exosomes have been found to be good carriers for drug delivery systems [12–14]. They can transport biologically active substances into the cytoplasm of immune cells or cancer cells and perform their biological functions precisely and efficiently [15, 16]. Exosomes have numerous advantages as therapeutic drug delivery vehicles, including a small size, good stability, and good biocompatibility and safety. Additionally, they are able to avoid phagocytosis by the reticuloendothelial system (RES) and penetrate deep into a tumor to release drugs by degrading the extracellular matrix [17].

**Fig. 1:**
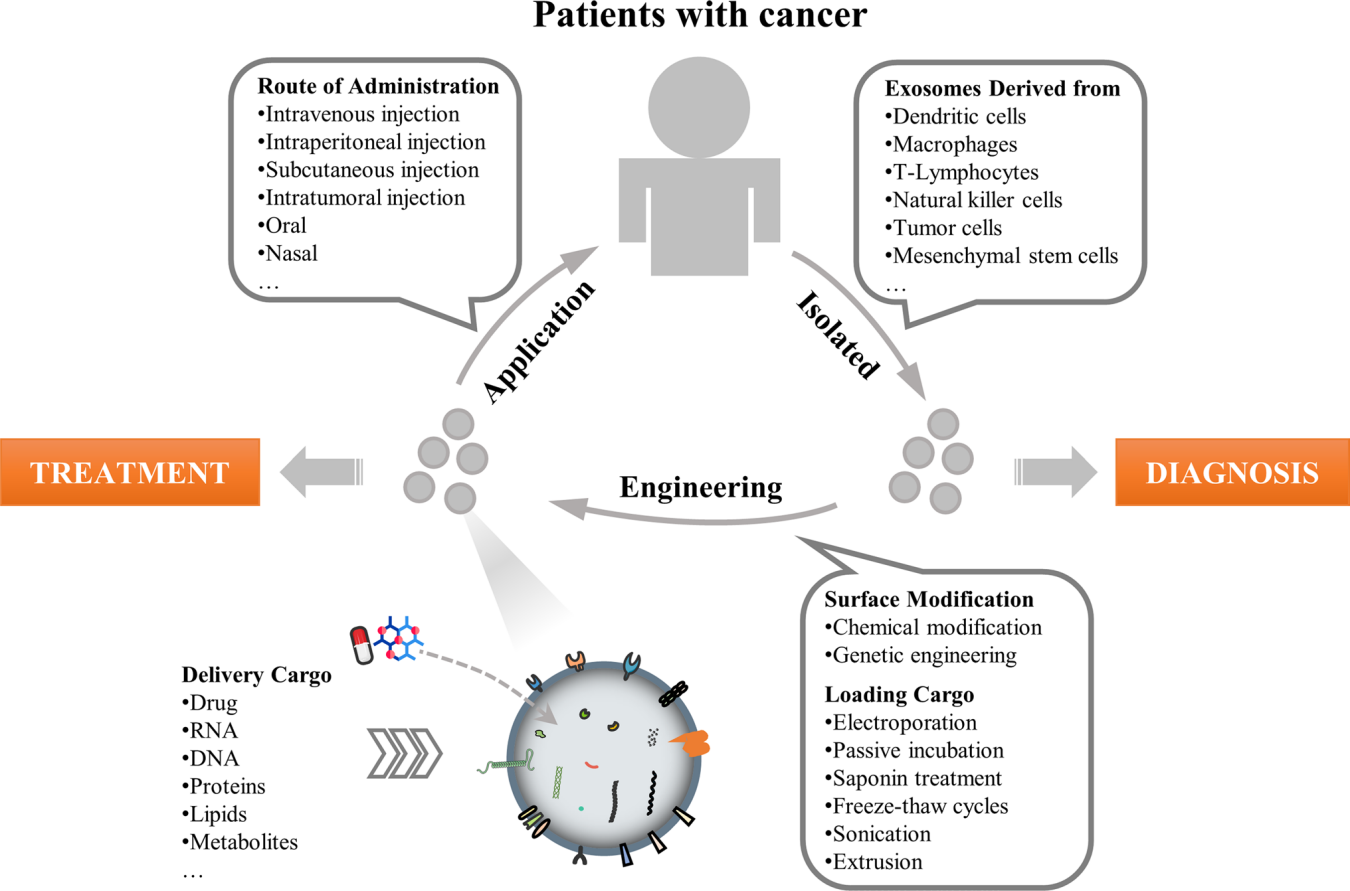
Exosome-based cancer diagnosis and treatment. Exosomes from different sources can be used for cancer diagnosis. Exosomes can be used as drug delivery systems for cancer treatment

The involvement of exosomes in cancer diagnosis and treatment is one of the current hotspots of cancer research (Fig. 1). An increasing number of studies have found that exosomes play an important role in the occurrence, development, metastasis, detection and treatment of cancer [18–24]. Comparing cancer cell exosomes to normal cell exosomes revealed that the levels of many of the proteins identified were particularly high in cancer exosomes. This is important because these markers can be used to diagnose exosomes from cancer cells and even identify which tissue they came from. For example, in 2015, a non-small cell lung cancer (NSCLC)-related study, which included blood sample data from 109 patients with stage IIIa-IV NSCLC and 110 controls, found that in the advanced NSCLC patients, CD317 and EGFR were highly expressed on the surface of exosomes [25]. Additionally, exosomes in the blood of treated patients can be monitored to understand the response to cancer treatment. If the exosomes decrease in number or disappear, it may indicate that the treatment is effective. If new mutations are found in exosomes, this could indicate that the cancer is developing new resistance to treatment. Potential tumor detection and treatment markers in exosomes in different tumors are described in detail in Table 2.

**Table 2:** Exosome markers for detection and treatment in different tumors

Tumors	Markers	Source	References
Breast cancer	miR-1910-3p, miR-20a-5p, TRPC5, Del-1, miR-200b-3p	Blood	[26–30]
Lung cancer	lncRNA UFC1, circ_0014235, BTG-1, miR-106b, circ_0001492, circ_0001439, circ_0000896, circ-MEMO1, lncRNA HOTAIR	Blood	[31–36]
Prostate cancer	lncRNA PCGEM1, Hyal1, PSA, miR-375, lncRNA AY927529, γ-glutamyltransferase (GGT), CD9, CD63, miR-142-3p, miR-142-5p, miR-223-3p, miR-342-3p, miR-374b-5p	Urine, Blood	[37–44]
Colorectal cancer	miR-200c-3p, miR-221/222, Dicer, miR-181d-5p, miR-6803-5p, miR-128-3p, circPACRGL, circ_0000338, miR-208b, miR-193a, let-7 g	Blood	[45–53]
Gastric cancer	miRNA-107, lncRNA GNAQ-6:1, lncRNA PCGEM1, Dicer, miR-423-5p, lncRNA pcsk2-2:1, CD63, DCLK1	Blood	[37, 54–59]
Liver cancer	circRNA 0006602, circRNA PDE8A, miR-221/222, miR-638, hnRNPH1, miR-92a-3p, miRNA-103, miRNA-224	Blood	[60–67]
Cervical cancer	CircEIF4G2, miR-663b	Blood	[68, 69]
Ovarian cancer	miR-124, PKR1, miR-200b, CD24, miR-21-5p	Blood	[70–73]
Esophageal cancer	Dicer, miR-320b	Blood	[46, 74]
Thyroid cancer	miRNA423-5p, Thyroglobulin	Urine, blood	[56, 75]
Bladder cancer	CA9, KRT6B, H19	Urine, blood	[76, 77]
Pancreatic cancer	PAK4, HIST2H2AA3, LUZP6,HLA-DRA, miR-19b, CD44v6, CD133, c-Met, PD-L1, CCT8, circRNA PDE8A, MMP14, Claudin7, Wnt5b, miR-191, miR-21, miR-451a, miR-30b-5p	Blood	[61, 78–88]
Leukemia	miR-10b, IFITM3, CD146, CD36	Blood	[89, 90]
Kidney cancer	CA9, miR-224-5p, γ-glutamyltransferase (GGT)	Urine, blood	[42, 91, 92]
Melanoma	CBP, miR-1180-3p, miR-222, PD-L1	Blood	[93–96]
Glioblastoma	circ-METRN, miR-253p, NANOG DNA	Blood	[97–99]

An emerging research area related to exosomes that has gained considerable attention is the application of exosomes in immunotherapy [12]. Exosomes derived from immune cells, tumor cells, and mesenchymal stem cells are the most widely used drug delivery systems [13, 100, 101]. Studies have confirmed that tumor-derived exosomes can carry drugs and target the drugs to tumor cells to inhibit their growth [14, 102–106]. Exosomes released by various immune cells (T cells, DCs, macrophages, etc.) play an important role in immune system regulation [107–110]. Immune cell-derived exosomes can mimic the characteristics of immune cells targeting tumor cells, conferring therapeutic benefits by attenuating or stimulating immune responses [13, 111]. Therefore, exosomes have great potential in cancer immunotherapy.

Although exosomes have been the subject of many review articles, few reviews have comprehensively summarized the role of exosomes as drug carriers in immunotherapy. In this review, we mainly focus on the application of exosomes for targeted drug delivery in cancer immunotherapy. The characteristics of exosomes as drug carriers and future prospects are also introduced.

## Exosomes as a drug delivery system

**Fig. 2:**
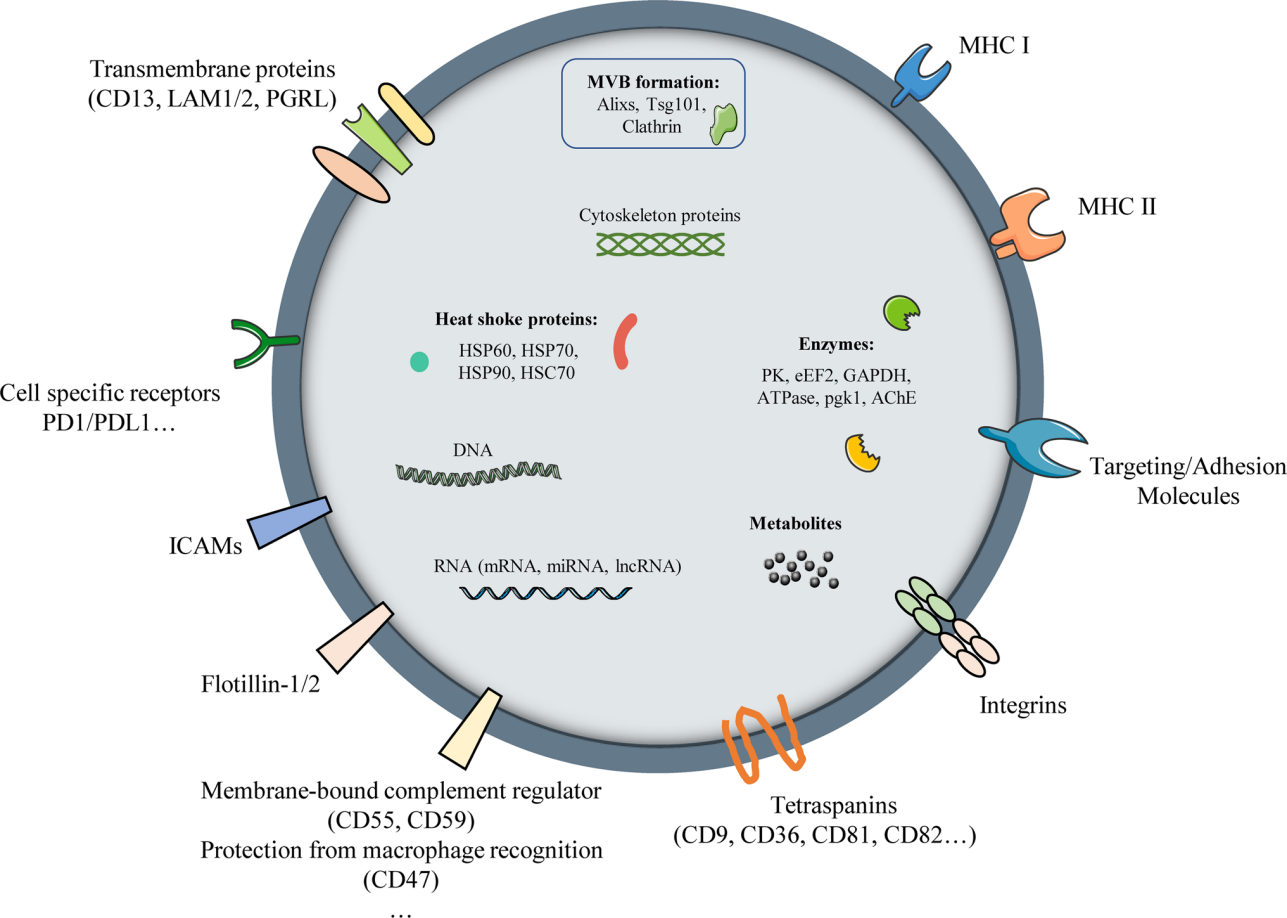
Schematic diagram of exosomes. The membrane and inside of exosomes carry a variety of proteins (CD13, LAM1/2, PGRL, PD1, PDL1, et al.), metabolites and nucleic acids (mRNA, miRNA, lncRNA, et al.)

Exosomes are widely found in various body fluids [107, 112]. In vivo, exosomes carry the membrane and cytoplasmic components of the parent cell and play the role of "courier", maintaining the exchange of substances (lipids, proteins, nucleic acids, etc.) between cells [113]. There are various membrane proteins with specific functions on the exosome membrane. For example, CD9 and CD81 help exosomes fuse with recipient cells, CD55 and CD59 protect against complement attack, and CD47 protects against phagocytosis by macrophages [114, 115] (Fig. 2).

Exosomes are naturally nontoxic and highly biocompatible, and they remain in blood circulation for long periods [116, 117]. These unique functions make exosomes a potential ideal drug delivery vehicle. However, autologous- and heterologous-dependent approaches need to be considered when choosing exosomes as drug delivery systems. The study found that the uptake of autologous and heterologous exosomes by recipient cells was significantly different. Autologous exosomes are more biologically similar to their parental cells and may be more suitable for drug delivery [118]. However, heterologous exosomes cannot be completely ignored. The acquisition of heterologous exosomes is often easier than that of autologous exosomes. Studies have found that heterologous exosomes can safely and reliably deliver drugs. Lessi et al. found that primary human macrophage-derived exosomes can be efficiently used for drug delivery [119].

In recent years, exosomes have been found to be a good drug delivery vehicle for cancer treatment [107, 120–122]. Several therapeutic approaches based on exosome drug delivery systems have entered clinical trials, as shown in Table 3.

**Table 3:** The ongoing clinical trials of exosome-based drug delivery systems for cancer therapy

ID	Study title	Cancer	Sources	Cargo	Phase
NCT01294072	Study investigating the ability of plant exosomes to deliver curcumin to normal and colon cancer tissue	Colon cancer	Plant exosomes	Curcumin	Phase 1
NCT03608631[123]	iExosomes in treating participants with metastatic pancreas cancer with KrasG12D mutation	Pancreas cancer	Mesenchymal stromal cells-derived exosomes	KrasG12D siRNA	Phase 1
NCT02657460	Clinical trial of tumor cell-derived microparticles packaging chemotherapeutic drugs to treat malignant pleural effusion	Malignant pleural effusion; advanced lung cancer	Tumor-derived microparticles	Methotrexate	Phase 2
NCT01854866	Safety and effectiveness study of tumor cell-derived microparticles to treat malignant ascites and pleural effusion	Malignant ascites and pleural effusion	Tumor cell-derived microparticles	Chemotherapeutic Drugs	Phase 2
NCT03230708	Clinical study of autologous erythrocytes derived MPs packaging MTX peritoneal perfusion to treat malignant ascites	Malignant ascites	Autologous erythrocytes-derived microparticles	Methotrexate	Phase 1/2

As a drug delivery system, exosomes also face numerous challenges and are affected by many factors. Prof. Gaurav, I. and Thakur, A. systematically reviewed the various factors affecting extracellular vesicle-based drug delivery systems [118]. For example, one issue is exosome isolation and yield. Although techniques for isolating exosomes have been widely reported and commercial extraction kits have been developed, current extraction techniques are still an important limiting factor in the application of exosomes for drug delivery [124]. There is no consensus on a standard procedure for the optimal isolation of exosomes. There are still insufficient technologies to obtain exosomes that can be used in drug delivery systems with high efficiency, high quality and low cost. In addition, exosomal surface modification is an important factor that affects targeted delivery [118]. Chemical modification and genetic engineering are two techniques that can be used for surface modification of exosomes. Surface modifications can affect the delivery capacity and biological effects of exosomes. However, neither is perfect. Due to the complexity of exosome surfaces, chemical modifications often lack site-specific control and even affect the structure and function of the carrier. Genetic engineering is the fusion of the gene sequence of the guide protein or polypeptide with the gene sequence of the selected exosomal membrane protein. This approach is effective for the surface display of polypeptides and proteins but is limited to genetically encoded targeting motifs.

In terms of drug loading, bioactive substances such as proteins, small RNAs and drugs can be loaded into exosomes using chemical methods and genetic engineering techniques [13, 102, 120]. At present, there are two methods to achieve drug loading in exosomes: endogenous loading and exogenous loading.

Endogenous loading, also known as preloading, refers to the loading of drugs into cells before the cells release exosomes. This method turns cells into living factories that release drug-loaded exosomes and directly secrete the desired drug-loaded exosomes. For example, Ran et al. transfected mouse embryonic fibroblasts (NIH3T) with a propeptide-expressing lentivirus (CD63-propeptide-expressing lentivirus), which ultimately enabled the fibroblasts to release exosomes carrying propeptides on their surface [125]. Choi et al. constructed a cell line that stably expressed two recombinant anti-inflammatory proteins, CIBN-EGFP-CD9 and srIκB-mCherry-CRY2. Then, irradiation with blue light (460 nm) induced cells to actively load anti-inflammatory proteins into exosomes [126]. The engineered cells can easily and conveniently produce the target exosomes, which have great potential in the commercialization of exosomal protein therapy. Fu et al. used genetic circuits to reprogram the host liver to direct the synthesis and self-assembly of siRNA into exosomes and facilitate the delivery of siRNA in vivo through circulating exosomes [127].

Exogenous loading, also known as postloading, refers to the processing of exosomes after isolation and purification of natural exosomes. There are various ways to load drugs into exosomes: coincubation, sonication, electroporation, freeze‒thaw, extrusion and permeabilization [16, 121, 128–137]. Thakur, A et al. successfully loaded two blood‒brain barrier (BBB)-impermeable anticancer drugs, DOX and PTX, into SF7761 stem cell-like GM-derived exosomes with an Exo-Load microfluidic device and found that this treatment exerted a strong tumor growth inhibitory effect [138]. Xu et al. found that exosomes secreted by M1 macrophages provide a proinflammatory environment and that paclitaxel (PTX) encapsulated by coincubation increases the antitumor ability of PTX in breast cancer cells through the caspase-3 pathway [139]. Prof. Alvarez-Erviti used electroporation to load exogenous siRNA into purified exosomes. Intravenous injection of exosomes carrying exogenous siRNA targeted for delivery to oligodendrocytes, microglia, and neurons in the brain results in specific gene knockout [140]. Li et al. used polycarbonate membrane extrusion to fuse drug-encapsulated nanoparticles with exosomes, and the cellular uptake efficiency and antitumor effect of doxorubicin (DOX) were significantly improved [13].

## Immune cell-derived exosomes

### Dendritic cells

Dendritic cells (DCs) are responsible for processing and presenting antigenic information in vivo [141, 142]. When DCs mature, they have many pseudopods similar to dendrites, so they are called dendritic cells [143]. Mature DCs specialize in processing and presenting various antigenic substances and play a central role in the immune regulation of the human body [144]. They can regulate the body’s humoral immunity, cellular immunity and tumor immunity. Through the processing of tumor cells, DCs can activate human T lymphocytes and enhance the phagocytosis of T lymphocytes on tumor cells, thereby exerting an effective antitumor effect [145]. DC-derived exosomes (Dexs) are able to enhance immune responses by transferring MHC complexes from antigen-exposed to unexposed DCs [111, 146]. These DCs load processed antigen onto major histocompatibility complex I and II (MHCI and MHCII) molecules, present to naïve CD8+ and CD4+ T cells, respectively, and transmit antigen memory to T cells [100]. Mature DCs are able to carry more intercellular adhesion molecule-1 (ICAM-1) and MHCII and exert stronger T-cell stimulation [147, 148].

In a phase II clinical trial, investigators used second-generation Dex (IFN-γ-Dex) for maintenance immunotherapy in patients with advanced non-small cell lung cancer (NSCLC). The study included 22 patients, and the primary endpoint was progression-free survival (PFS) 4 months after chemotherapy was stopped. This phase II clinical trial demonstrated the ability of Dex to enhance the antitumor immune response of NK cells in patients with advanced NSCLC [109]. Zhen et al. reported that alpha-fetoprotein (AFP)-expressing Dex could induce potent antigen-specific immune responses in ectopic or orthotopic hepatocellular carcinoma (HCC) mice, improved the immune microenvironment of autologous tumors, and decrease the amount of immune stimulation cell and CD8+ CTL infiltration, levels of immunosuppressive cytokines and the number of Treg cells [149]. A recent study reported that Dex vaccine (DEX_P&A2&N_) promoted the recruitment and activation of DCs in mice with liver cancer, thereby enhancing tumor-specific immune responses [150]. Studies have reported that DC cell-derived exosomes can cross the BBB to deliver RNAi to the brain and play a biological role, such as inhibiting tumor growth [151]. Xu et al. reported that fluorouracil could be encapsulated in DC cell-derived exosomes by electroporation and found that FU-DC-Exos had a strong inhibitory effect on the proliferation of colon cancer cells [152]. Using the property of rabies virus glycoprotein (RVG) to specifically bind to nicotinic acetylcholine receptors (AchR) on neurons and BBB vascular endothelium, Lakhal et al. established membrane-expressing LAMP2B-RVG exosomes (derived from DCs) for targeted delivery [151]. In conclusion, DC-derived exosomes offer great promise for cancer therapy as drug delivery vehicles.

### Macrophages

Macrophages are specialized, long-lived phagocytic cells of the innate immune system [153, 154]. They are the largest immune cell population in solid tumors and play an important role in maintaining homeostasis [155]. Macrophages have two main polarization states: the proinflammatory M1 phenotype and the anti-inflammatory and reparative M2 phenotype [156]. Macrophages can regulate their microenvironment and provide instructions to neighboring cells to maintain balance. Studies have found that macrophages can not only inhibit tumor growth and progression but also promote tumor cell growth, survival, and angiogenesis through an immunosuppressive microenvironment [156–158]. Feng et al. developed a macrophage-derived exosome-coated poly(lactic-glycolic acid) nanoplatform for targeted chemotherapy in triple-negative breast cancer (TNBC). This engineered exosome was found to have a significant tumor-targeting effect and to improve the cellular uptake efficiency and antitumor efficacy of doxorubicin [13]. M1 macrophage-derived exosomes (M1-exos) have been demonstrated to deliver anticancer drugs for cancer therapy. Kim et al. found that exosome membrane reorganization under the action of ultrasound could improve drug loading efficiency and sustained drug release. Therefore, the ultrasonic method was used to load PTX into M1-exos, and the results confirmed that M1-exos-PTX has a significant therapeutic effect on lung cancer [137]. Cianciaruso et al. found that macrophage-secreted exosomes have molecular features related to Th1/M1 polarization and enhance inflammatory and immune responses [159]. TAM-EVs also contain bioactive lipids and biosynthetic enzymes that may alter proinflammatory signaling in cancer cells. Therefore, although studies have found that macrophages can promote the malignant progression of tumors by stimulating angiogenesis, increasing tumor cell invasion and metastasis, and inhibiting antitumor immunity, the exosomes they secrete may stimulate rather than limit antitumor immunity [158, 159].

In conclusion, the potential of macrophage-derived exosomes for cell-to-cell communication in oncology research is unclear. Since macrophages are the largest immune cell population in solid tumors, the status and importance of macrophage-derived exosomes in future cancer research cannot be underestimated.

### T-lymphocytes

T lymphocytes are important immune cells in the body that fight diseases such as infections and tumors [160–162]. T lymphocytes include three types: natural killer T cells (NKT), T helper (Th) lymphocytes, and regulatory T cells (Tregs) [163]. Their functions include: (1) killing and eliminating virus-infected cells and cancer cells via cytotoxicity; (2) secreting cytokines to regulate the role of other immune cells; and (3) distinguishing exogenous pathogenic antigens and self-antigens to prevent inappropriate autoimmune responses. Qiu et al. found that PD-1 carried by T-cell-derived exosomes could interact with PD-L1 on the distal cell surface or exosomes. The internalization of PD-L1 is induced by endocytosis, preventing the binding of PD-L1 to PD-1 and thereby inhibiting the occurrence of immune escape [162].

Chimeric antigen receptors-modified T cells (CAR-T) have emerged as a promising new type of immunotherapy [164–167]. Johnson et al. used CAR-T cells to deliver RN7SL1, an endogenous RNA, to activate RIG-I/MDA5 signaling, stimulate a characteristic dendritic cell (DC) subset, and improve immune function [168]. Studies have found that CAR-T cells can release CAR-carrying exosomes and that CAR-expressing exosomes can significantly inhibit tumor growth, which may become a new antitumor therapy in the future [169]. Yang et al. found that exosomes derived from mesothelin (MSLN)-targeted CAR-T cells maintained the characteristics of parental T cells, such as CD3 expression on the membrane surface [170]. CAR-carrying exosomes can significantly inhibit the malignant progression of TNBC [170]. In conclusion, T-cell-derived exosomes are important mediators involved in immune regulation, and their application as drug delivery vehicles in cancer therapy is still in the exploratory stage.

### Natural killer cells (NK cells)

Natural killer cells (NK) are important immune cells that are related to antitumor and immune regulation [171]. NK cells can exert cytotoxic effects on a variety of cells, destroying infectious and tumor cells in the absence of antigen presentation [172]. NK-cell-derived exosomes (NK-Exos) contain the same molecules that kill cancer cells; they are much smaller than NK cells and are better able to penetrate tumors [173]. Exosomes secreted by NK cells also have a tumor-homing ability [110].

Kang et al. found that samples from patients with NSCLC contained more NK cells and NK-Exos, which were correlated with the number of circulating tumor cells (CTCs) [173]. CD56 and FLOT1 expressed by NK-Exos can be recognized and taken up by cancer cells, leading to cytotoxic death of cancer cells [173]. NK-Exos are also cytotoxic to melanoma cells and induce melanoma cell apoptosis. FasL inhibitors attenuate NK-Exos cytotoxic effects on melanoma. In vivo experiments in mice also showed that tumor size was significantly reduced after NK-Exos treatment [113]. NK-Exos have cytotoxic effects on tumors and good application prospects in cancer immunotherapy. miR-3607-3p in NK-Exos can target IL-26, thereby inhibiting the proliferation, invasion and migration of pancreatic cancer cells [174]. Taken together, these results show that NK-cell-derived exosomes are very promising in the field of tumor therapy, and the substances with antitumor effects that they carry are expected to become a promising new cancer treatment.

## Tumor cells-derived exosomes

Tumor-derived exosomes (TEXs) promote tumor growth and development in many ways, affect the differentiation and activation of immune cells and regulate antigen presentation [104, 175–178]. TEXs are key mediators of intercellular communication, can remodel distant microenvironments, such as premetastatic niches, and play an important role in the distant metastasis of tumors [106, 179, 180]. David et al. found that the tumor exosomal CEMIP protein can act on cerebral blood vessels and microglia, remodel the brain microenvironment, and promote the metastasis of cancer cells to the brain [181]. Tumor-derived exosomal miR-1247-3p directly targets B4GALT3, induces activation of the β1-integrin-NF-κB signaling pathway in cancer-associated fibroblasts, and promotes lung metastasis of liver cancer [104]. Studies have found that glioblastoma cell (GM)-derived exosomes can spread to systemic biological fluids through the BBB, which is considered to be an effective biomarker for the discovery of tracking glioma progression [8, 182]. GM-derived exosomes can penetrate the BBB, making it possible to deliver drugs that cannot penetrate the BBB to intracranial tumors. In addition, studies have reported that hypoxia increases the expression of MCT1 and CD147 in GMs, which leads to changes in the biological characteristics of exosomes released by GMs and affects the uptake of exosomes by receptor cells (e.g., endothelial cells) [182].

The diverse biological characteristics of TEXs make them effective molecular markers and therapeutic targets, especially for immunotherapy. Tumor cell-derived exosomal miR-21 and miR-29a can bind to TLR8 and TLR7 in immune cells, leading to the activation of NF-κB and the secretion of inflammatory factors [101]. NSCLC cell-derived exosomal circUSP7 suppresses CD8+ T-cell function by upregulating SHP2 expression by sponging miR-934, thereby promoting the resistance of NSCLC patients to anti-PD1 immunotherapy [105]. Samantha et al. found that primary tumor-derived exosomes can induce tissue-resident macrophages in the premetastatic microenvironment to upregulate the immunosuppressive molecule PD-L1 and secrete high levels of lactate, thereby establishing an immunosuppressive microenvironment that promotes tumor metastasis [176]. These research results confirm the regulatory effect of tumor-derived exosomes on the immune system and provide new targets for tumor immunotherapy.

Mauro et al. found that tumor-derived exosomes carrying PDL1 to lymph nodes can inhibit the function of T cells. Knocking out the TRAMP-C2 gene inhibited the release of exosomes from tumor cells, which in turn inhibited tumor growth. These results are in contrast to those obtained by the injection of exosomes carrying PDL1 collected in vitro [183]. Some studies have also found that tumor-derived exosome-loaded drugs can reduce the number of cancer cells [14, 102, 103]. This study confirms the important value of targeted inhibition of tumor-derived exosomes in tumor immunotherapy. In-depth exploration of the immunoregulatory mechanism of tumor-derived exosomes on various immune cells will help guide immunotherapy and overcome the resistance of current immune checkpoint inhibitors.

## Mesenchymal stem cells-derived exosomes

Mesenchymal stem cells (MSCs) are a type of pluripotent stem cell that have all the commonalities of stem cells, namely, self-renewal and multidirectional differentiation capabilities. MSCs exist not only in bone marrow but also in skeletal muscle, periosteum, and trabecular bone [184]. Mesenchymal stem cell-secreted exosomes (MSC-Exos) possess not only the tumor-regulating properties of parental cells but also the ability to transport valuable cargoes (such as proteins, lipids, RNAs) across physiological barriers to target cells and play a role in communication and regulation [185]. A recent study found that MSC-Exos can affect the occurrence and development of tumor cells by promotion or inhibition in two ways [186]. Wang et al. found that MSC-Exos could transfer miRNA-221 to HGC27 gastric cancer cells, thereby promoting the growth and migration of tumor cells [187]. Zhang et al. found that bone marrow mesenchymal stem cell-derived exosomes (BMSC-exos) carry miR-193a-3p, miR-210-3p and miR-5100 to recipient cells, activate the STAT3 signaling pathway to induce epithelial-mesenchymal transition, and enhance the invasive ability of lung cancer cells [188]. In addition, a study found that exosomal miR-145 derived from adipose MSCs inhibited prostate cancer growth by reducing Bcl-xl activity and promoting tumor cell apoptosis [189].

MSC-Exos have a certain targeting ability, and the modification of MSC-Exos can target the tumor site and have a stronger anticancer effect. Kamerkar et al. found that in a mouse model of pancreatic cancer, loading specific siRNA or shRNA carrying the oncogene KRAS into MSC-Exos significantly enhanced its efficacy and improved overall survival depending on CD47 [123]. Bagheri et al. used MSC-Exos as carriers to transport RNA, protein and small-molecule drugs to specific parts of the tumor for tumor therapy. For example, doxorubicin loaded into MSC-Exos by electroporation inhibited colon cancer growth. This mode of administration can significantly increase the accumulation of doxorubicin in tumor tissue [184, 190].

MSC-Exos still have considerable research potential and broad application prospects in the field of in vivo drug delivery, which can provide new research methods and ideas for cancer treatment. However, the research conclusions are still in the preclinical stage, and more in-depth basic research is needed to clarify its molecular mechanism in the future.

## Conclusions and prospects

In this review, we discuss the application value of exosome-based drug delivery systems, as well as the recent progress and application prospects of exosomes derived from immune cells, tumor cells and mesenchymal stem cells in the field of cancer immunotherapy. Exosomes can be released into the extracellular environment by immune cells or cancer cells. An increasing number of studies have shown that exosomes have important regulatory effects on the immune system [13, 107, 111]. Some biologically active substances on exosomes, such as MHC and costimulatory molecules, have been shown to be involved in exosome-mediated anticancer immune responses. A more comprehensive and in-depth understanding of the molecular mechanism of exosomes in immune regulation is of considerable importance for the development of anticancer immunotherapy based on exosome drug delivery systems.

Multiple studies have confirmed engineered exosomes to be an important tool for drug delivery, and multiple clinical trials are underway. For example, Thakur, A's team successfully loaded BBB-impermeable anticancer drugs into SF7761 stem cell-like GM-derived exosomes with an Exo-Load microfluidic device and inhibited tumor growth [138]. However, to facilitate the true application of exosomes in the clinic, there are still some hurdles that need to be further addressed. For example, exosome surface modification directly affects the efficiency of drug delivery. Although chemical modification and genetic engineering, two techniques that can be used for surface modification of exosomes, are widely used, both still have shortcomings. In the future, advances in exosome surface modification technology are crucial for the application of exosome-based drug delivery systems. In addition. isolating high-purity living NK-cell populations and extracting exosomes from these cells also face technical difficulties. At present, the extraction method of exosomes is mainly ultracentrifugation; however, the extraction yield is low, the cost is high, and it is difficult to achieve industrial production and large-scale clinical application [191]. Large-scale production and storage, biodistribution and heterogeneity, and engineered processing are all prominent challenges that must be overcome for clinical applications. At the same time, maintaining the stability and functionality of exosomes, the targeted therapeutic effects and the side effects of exosomes are also issues that must be considered.

In conclusion, we have made significant progress in understanding the biological properties of exosomes and their applications in the field of cancer over the past decade. Exosome-mediated drug delivery is expected to overcome important challenges in therapeutic areas, for example, drug delivery across biological barriers such as the BBB, and the use of patient tissue-derived exosomes as personalized and biocompatible therapeutic drug delivery vectors. However, there is still a long way to go before we can fully understand all the molecular mechanisms associated with exosomes and apply them in the clinic.

## Data Availability

Not applicable.

## Abbreviations

MHC: Major histocompatibility complex
ILVs: Intraluminal vesicles
MVEs: Multivesicular endosomes
ESCRT: Endosomal sorting complex
RES: Reticuloendothelial system
PTX: Paclitaxel
DCs: Dendritic cells
Dex: DCs-derived exosomes
ICAM-1: Intercellular adhesion molecule-1
NSCLC: Non-small cell lung cancer
PFS: Progression-free survival
AFP: Alpha-fetoprotein
HCC: Hepatocellular carcinoma
TNBC: Triple-negative breast cancer
M1-exos: M1 macrophage-derived exosomes
NKT: Natural killer T cells
Th: T helper
T regs: Regulatory T cells
CARs: Chimeric antigen receptors
MSLN: Mesothelin
NK: Natural killer cells
NK-Exos: NK cell-derived exosomes
CTCs: Circulating tumor cells
TEXs: Tumor-derived exosomes
MSCs: Mesenchymal stem cells
MSCs-Exo: Mesenchymal stem cells-secreted exosomes
BMSCs-exo: Bone marrow mesenchymal stem cell-derived exosomes
